# Genomic Epidemiology of SARS-CoV-2 in Pakistan

**DOI:** 10.1016/j.gpb.2021.08.007

**Published:** 2021-10-23

**Authors:** Shuhui Song, Cuiping Li, Lu Kang, Dongmei Tian, Nazish Badar, Wentai Ma, Shilei Zhao, Xuan Jiang, Chun Wang, Yongqiao Sun, Wenjie Li, Meng Lei, Shuangli Li, Qiuhui Qi, Aamer Ikram, Muhammad Salman, Massab Umair, Huma Shireen, Fatima Batool, Bing Zhang, Hua Chen, Yun-Gui Yang, Amir Ali Abbasi, Mingkun Li, Yongbiao Xue, Yiming Bao

**Affiliations:** 1 China National Center for Bioinformation, Beijing 100101, China; 2 National Genomics Data Center, Beijing Institute of Genomics, Chinese Academy of Sciences, Beijing 100101, China; 3 CAS Key Laboratory of Genome Sciences and Information, Beijing Institute of Genomics, Chinese Academy of Sciences, Beijing 100101, China; 4 University of Chinese Academy of Sciences, Beijing 100049, China; 5 CAS Key Laboratory of Genomic and Precision Medicine, Beijing Institute of Genomics, Chinese Academy of Sciences, Beijing 100101, China; 6 Department of Virology and Immunology, National Institute of Health, Islamabad 45500, Pakistan; 7 National Center for Bioinformatics, Programme of Comparative and Evolutionary Genomics, Faculty of Biological Sciences, Quaid-i-Azam University, Islamabad 45320, Pakistan; 8 Center for Excellence in Animal Evolution and Genetics, Chinese Academy of Sciences, Kunming 650223, China; 9 State Key Laboratory of Plant Cell and Chromosome Engineering, Institute of Genetics and Developmental Biology, The Innovation Academy of Seed Design, Chinese Academy of Sciences, Beijing 100101, China

**Keywords:** SARS-CoV-2, Virus, Molecular evolution, Haplotype network, Pakistan

## Abstract

COVID-19 has swept globally and **Pakistan** is no exception. To investigate the initial introductions and transmissions of the **SARS-CoV-2** in Pakistan, we performed the largest genomic epidemiology study of COVID-19 in Pakistan and generated 150 complete SARS-CoV-2 genome sequences from samples collected from March 16 to June 1, 2020. We identified a total of 347 mutated positions, 31 of which were over-represented in Pakistan. Meanwhile, we found over 1000 intra-host single-nucleotide variants (iSNVs). Several of them occurred concurrently, indicating possible interactions among them or coevolution. Some of the high-frequency iSNVs in Pakistan were not observed in the global population, suggesting strong purifying selections. The genomic epidemiology revealed five distinctive spreading clusters. The largest cluster consisted of 74 viruses which were derived from different geographic locations of Pakistan and formed a deep hierarchical structure, indicating an extensive and persistent nation-wide transmission of the **virus** that was probably attributed to a signature mutation (G8371T in *ORF1ab*) of this cluster. Furthermore, 28 putative international introductions were identified, several of which are consistent with the epidemiological investigations. In all, this study has inferred the possible pathways of introductions and transmissions of SARS-CoV-2 in Pakistan, which could aid ongoing and future viral surveillance and COVID-19 control.

## Introduction

Coronavirus disease 2019 (COVID-19) caused by SARS-CoV-2 created a severe public health crisis, globally affecting approximately 207 million people as of August 15, 2021 (worldometers.info/coronavirus/). It is known that infection, transmission, and mortality of infectious diseases are closely related to social environment such as population density, population mobility, and level of income [1], [2]. Pakistan, as the sixth most populous country, has a population of about 223 million with limited health care resources. Since the first COVID-19 patient in Pakistan was diagnosed on February 26, 2020 in Karachi [3], over half a million Pakistanis got infected till January 2021. Although stringent measures against COVID-19 were implemented in the middle of March, 2020, the number of infected cases kept growing and peaked in the middle of June 2020 (Figure S1). In Pakistan, Karachi and Lahore are the two most populous cities, while Punjab (PB) is the most densely populated province (source: www.Worldometers.info; Table S1). Besides, as a Muslim country (about 96.4% Muslim), religious gathering activities are frequent, which probably contribute to the spread of the disease. Fortunately, the number of newly diagnosed cases began to decline in late June, 2020, suggesting that novel control measures in Pakistan have played a positive and effective role. In August 2021, Pakistan experienced the fourth wave of infection with around 3000 new cases diagnosed per day.

Where the virus was introduced and how it was transmitted in the early stage of the epidemic in Pakistan are largely unknown. Genomic epidemiology is a powerful tool to answer these questions. However, only a few dozens of viral genomes from Pakistan were publicly released as of June 2020 [4], [5], [6], [7], [8]. To better understand the introductions and transmissions of SARS-CoV-2 in Pakistan, we sequenced oropharyngeal samples collected from March 16 to June 1, 2020 from 150 COVID-19 patients, with the viral nucleic acids enriched by a hybridization capture method. We analyzed nucleotide mutations, including intra-host single-nucleotide variants (iSNVs) and polymorphisms, which describe the viral difference within and between the hosts, respectively. iSNVs represent the intermediate stage between the origin and the fixation of the mutation at the individual level, while polymorphisms represent genome diversity at the population level. We further investigated the connections between the Pakistani sequences and the potential source of acquisition by putting the high-quality viral genomes into the context of the global diversity of SARS-CoV-2 genomes. These data and the genomic epidemiology analysis allowed us to understand the transmission dynamics and contribute to more targeted and effective response to the pandemic in Pakistan. Especially, it would aid in reinforcing the measures taken by the health sectors in Pakistan and worldwide to abate the risk of further spreading of the pandemic.

## Results

### Demographic details of the subjects

**Figure 1:**
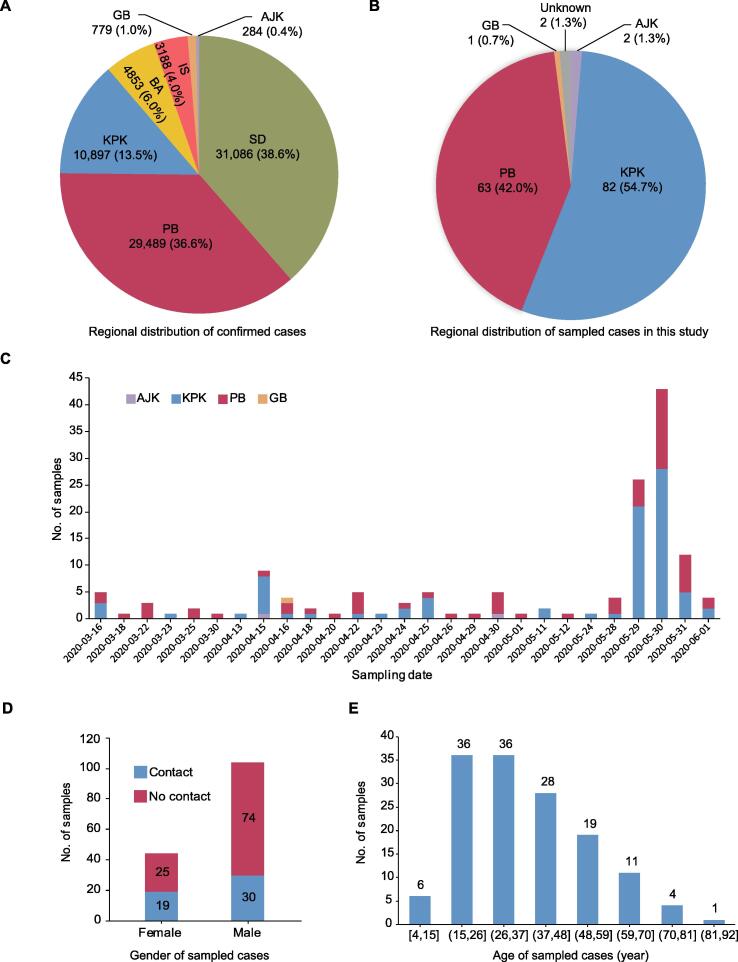
**Epidemic in Pakistan and demographic details of the confirmed COVID-19 cases sampled for sequencing in this study** **A.** Number of confirmed COVID-19 cases for Pakistan districts as of June 2, 2020. **B.** Regional distribution of 150 confirmed COVID-19 cases in Pakistan sampled for sequencing in this study. **C.** Number of samples on each sampling date from the districts indicated for the confirmed cases examined in this study. **D.** Sample distribution according to gender and contact history of cases in this population. Gender and contact information is missing for two different cases. **E.** Sample distribution according to age of the confirmed cases examined in this study. SD, Sindh; PB, Punjab; KPK, Khyber Pakhtunkhwa; BA, Balochistan; GB, Gilgit Baltistan; IS, Islamabad; AJK, Azad Jammu Kashmir.

From the distribution of confirmed cases (as of June 2, 2020) among administrative regions in Pakistan (Figure 1A), the highest numbers of infected individuals were found in Sindh (SD), PB, and Khyber Pakhtunkhwa (KPK), each with > 10,000 cases. Among them, 31,086 (38.6%) and 29,489 (36.6%) cases were from SD and PB (two provinces bordering India), respectively, while 10,897 (13.5%), 4853 (6.0%), and 779 (1.0%) cases were from KPK, Balochistan (BA), and Gilgit Baltistan (GB) that border Afghanistan, Iran, and China, respectively.

To improve our understanding of the introductions and transmissions of SARS-CoV-2 in Pakistan, in this study, oropharyngeal swab samples were collected from 150 confirmed COVID-19 cases (named E1–E150) in Pakistan from March 16 to June 1, 2020 by the National Institute of Health (NIH) in Pakistan. Most samples were collected from KPK (54.7%) and PB (42.0%) (Figure 1B), two provinces with severe epidemic (Figure 1A), while others were from Azad Jammu Kashmir (AJK, 2), GB (1), and unknown regions (2). Approximately 50% samples were collected on May 29 and May 30, 2020 (Figure 1C). Based on the epidemiological investigation, the male-to-female gender ratio was 104:44 (gender and contact information was missing for two different cases), and one third of these cases had contact history in this population (Figure 1D). The mean age of these cases was 37.6 ± 16.1 years old, which ranged from 4 to 85 years old (Figure 1E; Table S2). In general, the gender and age distributions of the cases examined were similar to those of early cases in Pakistan [3]. Notably, two cases had travel history, one (E120) back from Sweden and the other (E136) back from Qatar. The detailed epidemiological and clinical information for all cases is shown in Table S2.

### Genome sequences and variant analysis revealed Pakistan over-represented mutations

For all samples, we examined the Ct value of quantitative RT-PCR targeting SARS-CoV-2 and found it ranged from 16 to below detection limit, with a median of 24. Nucleic acids of SARS-CoV-2 were enriched by the probe hybridization method and sequencing was performed on Illumina Xten platforms. On average, 12 million paired-end reads were obtained for each sample, and a median number of 228,310 (range: 5163–358,458) reads per million (RPM) could be mapped to the SARS-CoV-2 reference genome (GenBank: MN908947.3). The median sequencing depth was 118,779 (range: 4896–769,788), and 94% of the samples (141 of 150) had more than 95% of the viral genome covered by at least 100-fold (Figure S2A; Table S2). Both the number of sequenced SARS-CoV-2 reads and the percentage of genome coverage were negatively correlated with the Ct value according to Spearman’s correlation coefficient (SCC) values (*r* = −0.45 and −0.61, respectively, *P* < 0.01; Figure S2B).

**Figure 2:**
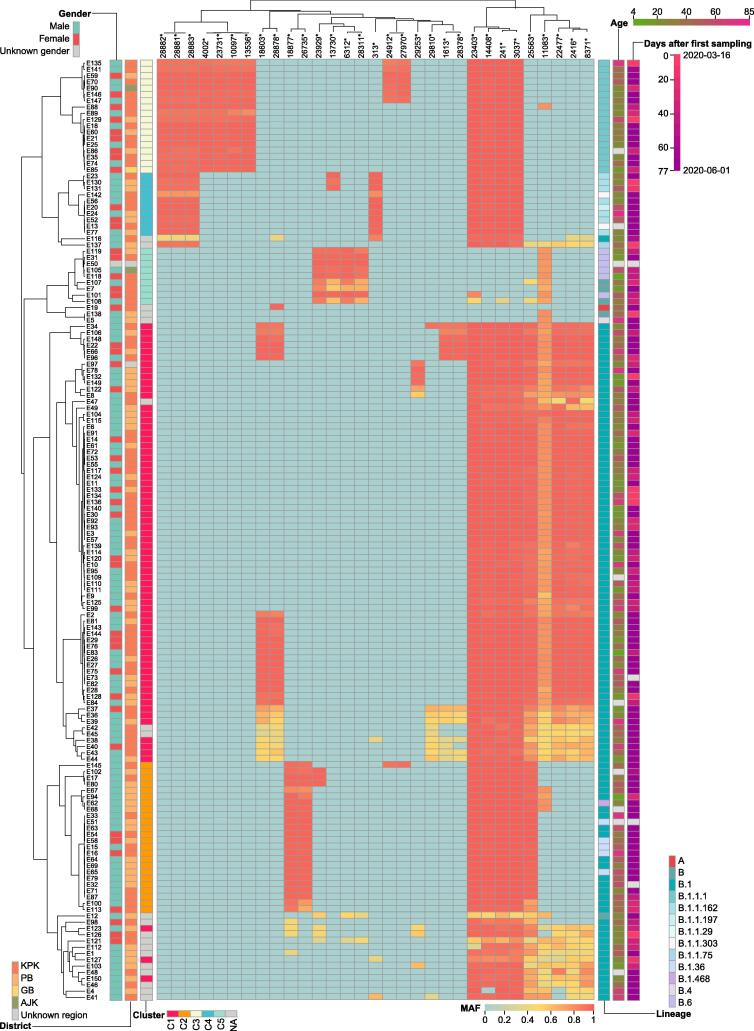
**Heatmap of MAF for variants with PMF > 0.05 in each sample** Accession ID of COVID-19 sampled cases is represented by a number prefixed with E (E is short for experiment). The gender, age, sampling date, and district information for each sampled case is shown with different color schemes. The Pangolin lineage and cluster information for each sample are also integrated. Variants that have significantly different (*P* < 0.05, Fisher’s exact test) PMF in Pakistani sequences compared to publicly-released sequences (as of October 9, 2020) are marked with asterisks. MAF, mutant allele frequency; PMF, population mutation frequency.

Both polymorphisms and iSNVs for each sample were identified by aligning sequenced reads to the reference genome (GenBank: MN908947.3). The number of polymorphic variants [with mutant allele frequency (MAF) > 0.3] ranged from 5 to 23, with a median of 12, which was slightly higher when compared to that of global publicly-released sequences (Figure S2C). In total, 347 mutation sites were identified, with 103 (29.7%) and 171 (49.3%) to be synonymous and nonsynonymous changes, respectively (Table S3). By calculating the population mutation frequency (PMF) for each variant, we found 31 variants with PMF > 0.05 (Figure 2), which means that they appeared in at least 8 samples. All of the 31 variants were over-represented (*P* < 0.05, Fisher’s exact test) in Pakistani sequences when compared to all publicly-released sequences (as of October 9, 2020). Consistent with the global trend [9], [10], a high frequency of the viruses (140, 93.3%) harbored the D614G amino acid change (conferred by a SNP at nucleotide 23403) in the spike protein. Besides, more than 30 Pakistani-specific mutation sites were detected, but with very low population incidence.

**Figure 3:**
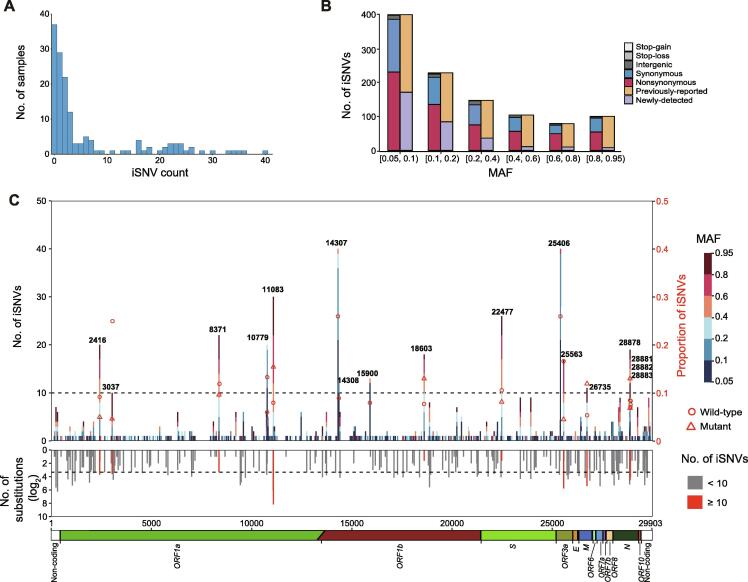
**Profile of iSNVs** **A.** Distribution of the iSNV count per sample. One sample with iSNV count of 109 is not shown in the bar chart. **B.** MAF distribution and mutation types of all iSNVs. Bars in orange and purple represent mutations that are observed and not observed in the polymorphism data (as of September 11, 2020), respectively. **C.** The number and genomic distribution of all iSNVs. In the top plot, the number of iSNVs at each position is plotted as a bar graph against the left Y axis, with MAF of each iSNV color-coded. The dash lines represent iSNV count of 10. Proportion of iSNVs for positions with iSNV count ≥ 10 is plotted against the right Y axis (major allele frequency ≥ 0.7, sequencing depth ≥ 100). Open circle and open triangle indicate wild-type and mutant nucleotides, respectively. In the middle plot, the grey histogram shows the substitution rate estimated from the polymorphism data. Positions with iSNV count ≥ 10 are indicated in red. In the bottom plot, the diagram shows the genomic structure of SARS-CoV-2. Coding regions are color coded with the respective gene names indicated below, and non-coding regions on both ends are shown in blank. iSNV, intra-host single-nucleotide variant.

As we observed some variants with low MAF in multiple samples, such as variants at positions 2416, 8371, or 11083 (Figure 2), we further detected 1057 iSNVs at 513 genomic locations (MAF > 0.05), including 599 nonsynonymous, 401 synonymous, 8 stop-gain, 1 stop-loss, and 48 intergenic mutations. The number of iSNVs per sample was unevenly distributed, ranging from 1 to 109 with a median of 3 (Figure 3A). The number of iSNVs was negatively correlated with MAF, except that in the range of 0.8–0.95, which may reflect back mutations (Figure 3B). We noted that 69.7% (737/1057) of the mutations were polymorphic in the population, and the iSNVs with higher MAF were more likely to be fixed in the population (*P* < 0.01, Fisher’s exact test). Positions with higher iSNV incidence (≥ 10) had higher substitution rates estimated from the polymorphism data (based on the information for 21,912 mutations in SARS-CoV-2 sequences from the 2019nCoVR database as of September 11, 2020) compared to the rest of iSNV positions (*P* < 0.01, Wilcoxon rank sum test). Meanwhile, the iSNV incidence was also positively correlated with the substitute rate (*ρ* = 0.22, *P* < 0.01, Spearman rank correlation). These data suggest that a large proportion of iSNVs with MAF > 0.05 were neutral or mildly deleterious, which did not undergo strong purifying selection during fixation in the individual. However, the two most mutable positions (14307 and 25406) and several highly mutable positions (10779, 14308, and 15900) were notable exceptions, since these mutations were rarely observed in the polymorphism data and had lower MAF (Figure 3C), probably reflecting a purifying selection or RNA editing at these positions. Of note, none of the sequence context of these mutations matched known RNA editing motifs. Furthermore, we noticed that iSNVs at position 11083 showed the highest MAF (mean MAF = 0.61) and this position also had the highest substitution rate among all positions, suggesting that either this position is error-prone during replication or the mutation at this position is favored by natural selection.

### Spread of SARS-CoV-2 in Pakistan revealed one large local transmission chain

**Figure 4:**
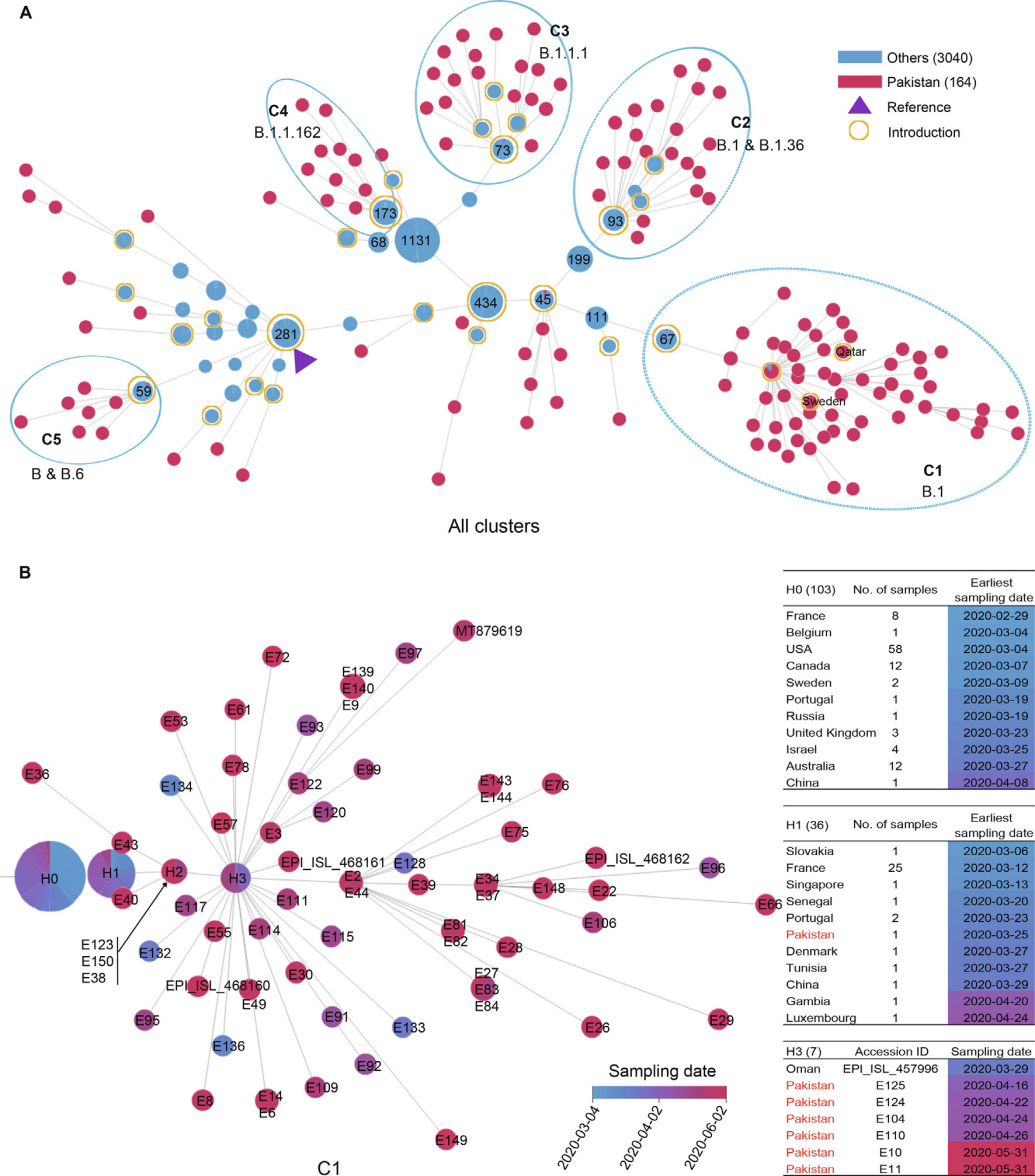
**Spread and transmission of SARS-CoV-2 sequences in Pakistan** **A.** Haplotype network of all SARS-CoV-2 sequences in Pakistan (Pakistan; red node) and closely-related publicly-released sequences from other countries (Others; blue node) as of October 9, 2020. Each node represents a distinctive haplotype, and the length of edge between any two nodes is proportional to sequence distance. Pakistani sequence clusters are labeled with C1–C5. Node of the reference sequence is marked by a solid triangle in purple, and nodes of putative introductions are labelled with open circles in yellow. Number of samples for public sequences are marked in each node when available. **B.** The haplotype network of C1. The color of the nodes, from blue to red, represents the sampling date for SARS-CoV-2 sequences in Pakistan as shown for (A) from March 4, 2020 to June 2, 2020. Sample accession ID is marked in each node. Nodes marked by H0, H1, and H2 represent the parent, the first, and the second generations of Pakistani sequences, respectively. Node of H3 indicates the super spreader sequences. Number of samples from different countries for H0 and H1, as well as sample details for H3 are listed in the table on the right.

To understand the spread and transmission of SARS-CoV-2 in Pakistan, we performed both haplotype network and phylogenetic analyses (Figure S3) using the 150 newly obtained SARS-CoV-2 genomes from Pakistan in this study along with 14 published Pakistani sequences and 2135 randomly sampled high-quality global publicly-released sequences (from 54,204 sequences with the collection date before June 17, 2020) as of October 9, 2020. Multiple local clusters were observed in both analyses. As haplotype network can reflect the variety of transmission possibilities of the virus and can clearly reflect the transmission relationship between virus strains [11], the sequences closely related to Pakistani sequences in the network (including the parental and filial sequences of the Pakistan nodes) were further selected to infer the virus transmission chain (Figure 4A). For all Pakistani sequences, we identified 140 distinct haplotypes (Table S2), and most sequences can be further grouped into five distinct clusters, defined as C1–C5, with 9–74 sequences each.

The largest cluster (C1) included 74 Pakistani genomes sampled from March 16 to June 2, 2020, and was characterized by a nucleotide substitution G8371T in *ORF1ab*, causing an amino acid change of Q2702H in *ORF1ab* (Table 1). Nearly all sequences in C1 could be classified as the B.1 lineage based on Pangolin COVID-19 Lineage Assigner [12]. Here, we made a few interesting observations. First, the common ancestor sequence of C1 (node marked as H1, having the lowest number of variants compared to reference) consisted of one Pakistani sequence (E127, collected on March 25, 2020 in PB) and 35 sequences from France, Slovakia, Portugal, Singapore, Senegal, *etc*. (Figure 4B). And the sampling date for majority of these publicly-released sequences was earlier than that of Pakistani sequence. By further tracing the upstream parent sequences (H0) of this Pakistani sequence, we found that the sampling date of some sequences from USA, France, Canada, Belgium, and Sweden was also earlier than sample E127 and the date when Pakistan began to suspend international flights (March 13, 2020), suggesting that this Pakistani sample might be related to early international introductions. Secondly, another key node (marked as H3 in Figure 4B) consisted of seven sequences, six from Pakistan and one from Oman. The haplotype network analysis showed that the H3 node had more filial nodes, suggesting these six Pakistan cases may be super spreaders in the transmission chain. As the sampling date of Oman sequence (March 29, 2020) was earlier than that of the six Pakistani sequences (April 16–May 31, 2020), we speculated that these infected cases in Pakistan could be Oman importation related. Thirdly, there were four descendent sequences (E132, E133, E134, and E136) that sampled earlier than Pakistani sequences in the H1 and H3 nodes. Among them, E136 was collected from Federal Capital on March 16, 2020 from a case who had a travel history to Qatar. These four cases might also be imported from abroad. Fourthly, nearly all C1 sequences carried the common signature nucleotide mutations (C2416T, C3037T, G8371T, C14408T, A23403G, and G25563T), which formed a specific haplotype, and these descendent sequences were mainly Pakistan-specific, forming at least 2–5 transmission chains, indicating that viruses in C1 were mutated and widely transmitted in Pakistan. Moreover, most cases of C1 were collected from PB (*n* = 30) and Federal Capital (*n* = 38), two adjacent districts. The local transmission across the country could be the result of expansion of cases seeded by the local religious congregations in Lahore (the capital of PB) and social gatherings and later by relaxing the lock-down situation.

**Table 1:** **Major clusters and signature variants of SARS-CoV-2 genome sequences in Pakistan**

**Parameter**	**C1**	**C2**	**C3**		**C4**	**C5**	
Lineage	B.1	B.1; B.1.36	B.1.1.1		B.1.1.162	B; B.6	
No. of sequences (this study/publicly-released sequences as of October 9, 2020)	74 (70/4)	24 (24/0)	22 (18/4)		10 (10/0)	9 (9/0)	
Common variants	C2416T, C3037T, G8371T, C14408T, A23403G, G25563T	C3037T, C14408T, C18877T, A23403G, G25563T, C26735T	C3037T, C4002T, G10097A, C13536T, C14408T, A23403G, C23731T, G28881A, G28882A, G28883C		C313T, C3037T, C14408T, A23403G, G28881A, G28882A, G28883C	C6312A, G11083T, C13730T, C23929T, C28311T	
Putative or likely importing countries	**USA**, **France**, **Belgium**, **Canada**, **Sweden**, ***Oman***, ***Qatar***	**Japan**, **Saudi Arabia**, **Turkey**, India, Indonesia, Kazakhstan	**UK**, Austria, Costa Rica, Czech Republic, Denmark, Israel, Netherlands, Peru, Portugal, Sweden		Brazil, Denmark, France, Hungary, India, Israel, Japan, Norway, Portugal, Russia, Singapore, Republic of Korea, Switzerland, United Arab Emirates, UK, USA	USA, Australia, Canada, China, Gambia, India, Malaysia, New Zealand, Oman, Senegal, Sierra Leone, Singapore	
No. of putative introductions	4	3	4		1	1	
Signature variant	G8371T	C26735T	C4002T	C13536T	C313T	G11083T	C23929T

Gene containing the signature variant	*ORF1ab*	*M*	*ORF1ab*	*ORF1ab*	*ORF1ab*	*ORF1ab*	*S*
Amino acid change for the signature variant	Q2702H	−	T1246I	−	−	L3606F	−

*Note*: Putative and likely importing countries for each cluster are presented in bold and regular fonts, respectively; countries visited by two patients are presented in italic. Signature variant refers to the mutation present in each cluster but absent from its parental nodes. “−” indicates synonymous mutation that does not cause amino acid change.

### Inferred multiple introductions and international transmission routes

The haplotype network analysis also revealed four clusters (C2–C5) that did not form a large transmission chain which may be due to Pakistan's local epidemic prevention and control policy.

**Figure 5:**
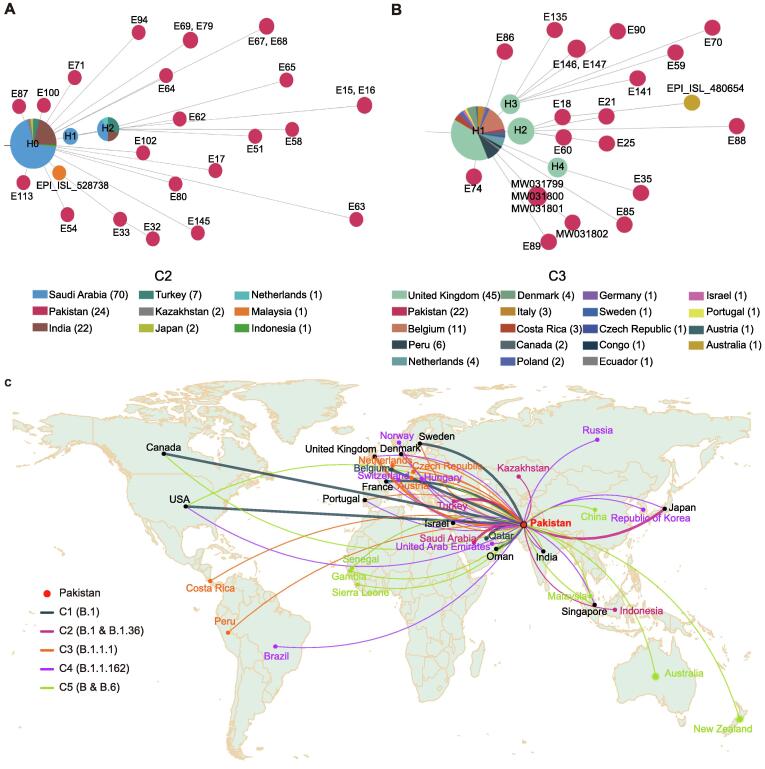
**Two representative introduction-related clusters and schematic diagram of inferred international importing routes** **A.** Haplotype network of C2. **B.** Haplotype network of C3. **C.** Schematic diagram of inferred global introductions. Thicker lines represent putative importing countries and thin lines represent other likely importing countries.

C2, assigned as the B.1 and B.1.36 lineages, had 24 Pakistani sequences which were characterized by a synonymous nucleotide substitution (C26735T) in the *M* gene. Aside from this synonymous mutation, all C2 sequences carried other five mutations: C3037T, C14408T, C18877T, A23403G, and C25563T. No identical sequences to any of the ones in C2 were found in public databases. To find the possible source of introductions of viruses in C2, the upstream parental node (H0) of C2 Pakistani sequences were scrutinized. There were 93 sequences sampled from six Asian countries in H0 node, including India, Indonesia, Japan, Turkey, Kazakhstan, and Saudi Arabia (Figure 5A), and three derived nodes consisted of 13 sequences collected from Malaysia, Saudi Arabia, Turkey, India, and the Netherlands. More importantly, the sampling date of 46 sequences from Japan, Saudi Arabia, and Turkey was earlier than that of Pakistani sequences in C2 and the flight suspension date of Pakistan. We therefore infer that there may be most probably three independent international introductions to Pakistan from Japan, Saudi Arabia, and Turkey.

There were 22 Pakistani sequences (18 generated from this study and 4 collected from public databases) in C3, which were designated as the B.1.1.1 lineage [12]. C3 was characterized by two nucleotide substitutions (C4002T and C13536T) in the *ORF1ab* gene, and the former caused an amino acid change T1246I. In total, 13 sequences in C3 were directly connected to sequences from the United Kingdom (UK), while the remaining 9 sequences were associated with those spread in countries including Austria, Costa Rica, Czech Republic, Denmark, Israel, Netherlands, Peru, Portugal, and Sweden (Figure 5B). According to the haplotype network analysis, we speculate that there may be at least four international introductions.

C4 contained 10 Pakistani sequences and was designated as the B.1.162 lineage. When tracing the parental node of C4 sequences, we found 173 sequences that were distributed in nearly 20 countries, such as Denmark and France, suggesting that the source of C4 cases in Pakistan could be complex. Based on the haplotype network analysis, we speculate that there may exist one or more independent international introductions in C4 (Figure 4A). For C5, there are 9 Pakistani sequences that can be traced back to a sequence sampled on March 20, 2020 in USA. The descendant sequences were spread in more than 10 countries by adding two additional mutation sites (G11083T and C23929T), including 2 Pakistani samples (E108 and E118) collected in the middle of April, 2020. So, we infer that they might be introduced from USA, Australia, Canada, Guangdong/Taiwan of China, Gambia, India, Malaysia, New Zealand, Oman, Senegal, Sierra Leone, or Singapore.

In all, by tracing the parental nodes of all clusters using haplotype network analyses, we infer several putative importing routes (thicker line in Figure 5C), including USA, UK, France, Sweden, Japan, Saudi Arabia, and Turkey, and multiple likely introductions (thin line in Figure 5C). Based on the number of network edges, we infer that there may be 28 introductions. To further test the likelihood of early introductions, we compared the beginning date of epidemic between Pakistan, its neighboring countries, and the putative importing countries. We found that most countries had outbreaks earlier than Pakistan, except for Saudi Arabia, Qatar, and Turkey (Figure S4).

## Discussion

This is the largest genomic epidemiology study of COVID-19 in Pakistan, with 150 high-quality complete SARS-CoV-2 genomes obtained and analyzed. SARS-CoV-2 evolves at a rate of approximately 1–2 mutations per month [13]. Hundreds of mutation sites and 1057 iSNVs were identified, some of which were found over-represented in this cohort, suggestive of a wide spread in Pakistan. High sequence diversity was also reflected by 128 different haplotypes among 150 sequenced genomes. On the one hand, this may be due to a long sampling date span, and most of the samples were collected at the peak of outbreak. On the other hand, it may indicate that there are continuous importations of different virus types.

An unprecedented sequencing depth was obtained in this study for SARS-CoV-2 genome due to the implementation of an efficient hybridization enrichment method, which enables not only the detection of the mutations, but also the analysis of the iSNVs. iSNV could represent either *in vivo* evolution of virus after infection or infection of different virus strains simultaneously or successively. Although we identified several mutations that tend to occur concurrently and showed similar frequencies, they cannot be explained by coinfection of a secondary strain that was included in our dataset, since no single sequence in our dataset contains all these mutations. We suspect that these concurrent mutations may be explained by strong compensatory effects of epistasis [14], [15]. The long tail in the distribution of iSNV count in each sample agrees with several recent findings [16], [17], and the fast evolution of the virus in some patients may result in the generation of new strains with excessive number of mutations [18], [19]. Most iSNVs occurred at positions with high substitution rate, suggesting that a large fraction of mutations may be neutral despite that SARS-CoV-2 genome evolves under purifying selection (File S1) [17], [20]. In contrast, we noticed that the two most frequently observed iSNVs (T14307C and T25406C) in our data were located at positions that were not polymorphic at the population level. These findings suggest that these iSNVs could be either dysfunctional mutations subjected to intensive purifying selection or caused by *in vivo* modification, such as RNA editing. However, these iSNVs were not located in the sequence context known to be recognized by APOBEC or ADAR protein families. Besides, our results indicate that some mutations shared by different cases were indeed iSNVs, which might arise independently after infection. How iSNVs could be used for genome epidemiology warrants further investigation.

The mutations detected in the SARS-CoV-2 sequences from Pakistan could be used to infer the source and transmission chain of SARS-CoV-2 within the country [21]. Furthermore, such data can also be helpful to monitor the export of virus to other countries. Based on the phylogenetic and haplotype network analyses, we infer about 28 independent introductions during the 3-month study period. Notably, viruses in one dense cluster C1 had been widely transmitted in the community, spanning KPK and PB regions. The fast spread of the epidemic may be due to a mass religious gathering in Lahore (the capital of PB) held during March 11–12, 2020. It has been reported that COVID-19 transmissions are often associated with clusters of cases linked to gatherings, including those in work places [22], churches [23], and especially in group living environments such as care homes and homeless shelters [24]. These social gathering-based viral clusters are thought to often involve superspreading [24].

Nearly all C1 sequences (73/74) were directly or indirectly linked to several Pakistani sequences in the H1 and H3 nodes, and formed a transmission chain of up to five generations. To better control the epidemic in Pakistan, any social gatherings whatsoever should be strictly controlled. On the other hand, the fast transmission ability of C1 virus might be attributed to its characteristic nucleotide mutation G8371T in the coding region for nonstructural protein 3 (nsp3) in *ORF1ab*. To test this possibility, we collected all publicly-released sequences (as of February 22, 2021) with the G8371T mutation and built a haplotype network (Figure S5). Several local clusters spreading with multiple generations were found, suggesting that this mutation may enhance viral transmission. nsp3 is a predicted phosphoesterase which is a component of the replication–transcription complex (RTC), and the interaction of N and nsp3 proteins is essential for viral genome processing. Some residues involved in the interaction between nsp3 and the C-terminal domain (CTD) of N protein have been predicted, which could be a potential drug target as inhibitors [25]. Moreover, 188 mutations (1–9 per isolate) occurred after the introduction of the two virus isolates (H1 and H3 nodes) in Pakistan. Whether these mutations alone or in combination could increase the transmissibility of the virus warrants further investigation.

Based on our haplotype network tracing analysis, the early epidemic in Pakistan was related to the aforementioned international introductions, as the parental nodes of all clusters and those sporadic cases were directly connected with virus sequences outside Pakistan. For example, the parental node sequences of C1 containing C2416T/C3037T/C14408T/A23403G/G25563T was reported to be associated with an international business conference in Boston, resulting in extensive international spread and low-level community transmission in Europe [26]. The C2416T/G8371T sub-lineage was likely the first in the C1 cluster imported into Pakistan based on the most recent common ancestor (MRCA) estimation — the median estimated date to MRCA of the C1 cluster was March 10, 2020 (95% CI: March 7–14, 2020) [13]. The mutation appeared frequently among SARS-CoV-2 genomes from Europe and USA in public databases (81 genomes as of February 19, 2021), suggesting its introductions from USA or Europe. Genomes of another sister cluster C2, harboring the C26735T variant in addition to common signatures of C3037T/C14408T/A23403G/C18877T/G25563T, have been found mainly in Asia (93 genomes as of October 9, 2020). The other two clusters, C3 and C4, were derivatives from European lineages B.1.1.1 and B.1.1.162, respectively. Furthermore, viruses of the C1, C3, and C4 clusters were initially identified before travel bans and they continued to spread in country. Although Pakistan closed its land borders and limited international flights in the middle of March, 2020, viruses in C2 and C5 directly related to the ones outside the country have continued to be isolated in Pakistan and spread in small scale. This reveals that viruses might be imported into Pakistan through other routes, such as cold-chain transportation in the frozen food industry [27]. It is therefore important for Pakistan to strengthen the inspection and quarantine of imported cold-chain food and pay more attention to the personal protection of workers involved to better prevent and control COVID-19.

In summary, we have generated a total of 150 high-quality and complete SARS-CoV-2 genomes of Pakistan, and identified 31 over-represented mutations. Our genomic epidemiology study reveals five distinctive spreading clusters. Among them, C1 was the largest cluster and introduced putatively from USA, France, Belgium, Canada, Sweden, Oman, and Qatar. The deep hierarchical structure of C1 indicates an extensive and persistent nation-wide transmission of the virus, which is probably attributed to a signature nucleotide mutation G8371T in the *ORF1ab* gene. Besides, more than 20 putative or likely international introductions were inferred from Europe and the Middle East, and several of them were consistent with the result of epidemiological investigation. However, these findings are also limited due to the limitation in the locations and date period for sampling.

## Materials and methods

### Sample collection

Confirmed COVID-19 cases were from sentinel sites, secondary and tertiary care hospitals, and high risk area of COVID-19 transmission in Pakistan. Predesigned forms were used to collect information on their demography, clinical history, and risk factors according to the WHO definition [28]. Throat and/or nasopharyngeal swab samples were collected from the subjects in 2–3 ml of viral transport medium (Virocult) and stored at −80 °C.

### RNA extraction and preparation

RNA was extracted using Qiagen QIAmp Viral RNA Mini Kit (QIAGEN, Hilden, Germany), eluted in 60 μl of elution buffer, and analyzed by one-step real-time RT-PCR on Applied Biosystems platform ABI 7500 (ThermoFisher Scientific, Foster City, CA) following the protocol recommended by WHO [29]. The assay was performed using AgPath-ID One-Step RT-PCR Kit (Ambion, Carlsbad, CA). Briefly, 25 μl of PCR mixture containing 0.5 μl each of probe (FAM-labeled), forward and reverse primers, 1 μl of enzyme mix, 12.5 μl of 2× master mix, 5 μl of nuclease-free water, and 5 μl of extracted RNA was subjected to amplification with the following conditions: reverse transcription at 55 °C for 10 min, Taq enzyme activation at 95 °C for 3 min, and 45 cycles of 95 °C for 15 s and 58 °C for 30 s. Inactivated RNA samples on dry ice were sent to China National Center for Bioinformation (CNCB) by air.

### Metatranscriptome library preparation

The concentration of SARS-CoV-2 RNA samples was quantified with RT-qPCR targeting the *ORF1ab* gene (Real-Time Fluorescent RT-PCR Kit for Detecting 2019-nCoV, BGI, Shenzhen, China). Total nucleic acid was concentrated using RNA Clean & Concentrator-5 Kit (Zymo Research, Irvine, CA) based on the manufacturer-supplied protocol. The concentrated samples were converted into Illumina-compatible sequencing libraries with Trio RNA-seq Kit (Nugen, Redwood City, CA). First, DNA leftover was digested through DNase treatment. After first-strand cDNA was synthesized, 8 μl of cDNA was used for multiplex PCR sequencing, and 10 μl of H_2_O was added to the remaining 15 μl of product for the second-strand cDNA synthesis. Single primer isothermal amplification (SPIA) was applied to amplify cDNA. Resultant DNA was subjected to fragmentation, end repair, and adaptor ligation through 6 cycles of PCR amplification. The DNA fragments bound to AnyDeplete probes were discarded to reduce subsequent amplification of human rRNA. Finally, 8 cycles of PCR were applied, and the product was purified with AMPure XP beads (Beckman, Brea, CA).

### Hybrid capture-based enrichment and sequencing

A 750-ng input from the library was used for hybrid capture-based enrichment of SARS-CoV-2 with two rounds of hybrid capture-based hybridization (TargetSeq One nCov Kit; iGeneTech, Beijing, China). Each enrichment was carried out based on manufacturer’s instructions using a hybridization temperature of 65 °C for 16 h and followed by 22 cycles of PCR in the first round, and 14 cycles in the second round. Sequencing was performed on Illumina HiSeq Xten platform (San Diego, CA) with 32 samples multiplexed in a lane.

### Sequence read preprocessing and variant calling

Sequencing reads were preprocessed by eliminating low-quality bases (Q20 < 10), adapter sequences, and reads with length < 30 bp using Cutadapt [30]. High-quality reads were mapped to the reference genome of SARS-CoV-2 (GenBank: MN908947.3) using Burrows-Wheeler Aligner (BWA) [31]. Reads with high mapping quality (MQ > 25) were retained by SAMtools [32], and duplicated reads were marked with MarkDuplicates package in Genome Analysis Toolkit (GATK) [33]. Genomic variants were identified using uniquely-mapped reads by HaplotypeCaller package in GATK. The genotype was assigned as a mutant allele if the frequency of mutant allele is ≥ 0.7, and a degenerate nucleotide was assigned if the frequency of mutant allele was < 0.7 and ≥ 0.3; the reference allele was assigned otherwise. Effect of variants (MAF > 0.3) on genes, transcripts, and protein sequences, as well as regulatory regions was annotated by Ensembl Variant Effect Predictor (VEP) [34]. The depth of sequencing and the coverage of genome were calculated based on the high-quality mapped (HQM) reads without duplications.

### Detection of iSNVs

To identify the iSNVs, mpileup files were generated by SAMtools v1.8 and then parsed by VarScan v2.3.9 along with an in-house script. All identified iSNVs have to satisfy the following criteria: 1) sequencing depth ≥ 100, 2) minor allele frequency ≥ 5%, 3) minor allele frequency ≥ 2% on each strand, 4) minor allele count ≥ 10 on each strand, 5) strand bias of the minor allele < 10, 6) minor allele being supported by the inner part of the read (excluding 10 bp on each end), and 7) minor allele being supported by ≥ 10 reads that were mapped exclusively to the genome of betacoronaviruses by Kraken v2.0.8-beta on each strand.

For comparison with the polymorphism in the population, we obtained the information for 21,912 mutations in SARS-CoV-2 sequences from the 2019nCoVR database (https://ngdc.cncb.ac.cn/ncov/variation) [6], [8] from CNCB-National Genomics Data Center (CNCB-NGDC) as of September 11, 2020.

### Substitution rate estimation

To estimate the substitution rate at each position, SARS-CoV-2 genome variations and corresponding metadata were downloaded from 2019nCoVR (https://ngdc.cncb.ac.cn/ncov/variation) [6], [8]. Phylogeny and ancestral sequences were constructed by IQtree (v2.1.1), using the reference sequence (GenBank: MN908947.3) as the outgroup of the phylogenetic tree. Substitution on each branch was inferred by comparing to its parental node. The substitution rate of each position was represented by the sum of the substitution events observed on the phylogenetic tree.

### Genome assembly construction

The consensus sequence for each sample was built based on the reference sequence and filtered variants using in-house Perl scripts. Specifically, the bases of mutation sites in the reference genome were replaced with their altered alleles. When sequence variation is heterozygous, the base is marked using a degenerate base symbol. And, if the mapping depth of a base is < 30, the base is considered to be an unknown base and marked as N.

### Haplotype network construction and phylogenetic analysis

Global sequences including 14 Pakistani sequences were downloaded from the 2019nCoVR database in CNCB (https://ngdc.cncb.ac.cn/ncov) [6], [8] and the global initiative of sharing all influenza data (GISAID) EpiCoV database [35], [36] as of October 9, 2020. Among them, 54,204 sequences sampled before June 17, 2020 were used for further analysis. Along with the 150 Pakistani SARS-CoV-2 sequences from this study, haplotype network was constructed as in the 2019nCoVR [8]. Briefly, short pseudo sequences that consist of all variants (excluding those in the UTRs) were generated for all 54,354 sequences. All these pseudo sequences were clustered into groups, and each group (a haplotype) represents a unique sequence pattern. The haplotype network was inferred from all identified haplotypes, where the reference sequence haplotype was set as the starting node, and its relationship with other haplotypes was determined according to the inheritance of mutations. Java Server Pages (JSP), HTML, and D3.js (a JavaScript library for manipulating documents based on data; https://d3js.org) were employed to visualize the haplotype network. In order to show the transmission of SARS-CoV-2 in Pakistan more clearly, the sub-network directly related to Pakistani sequences was constructed. Clusters were defined based on the network topology. The lineage for each sequence was also determined by Pangolin (v2.0.8, Phylogenetic Assignment of Named Global Outbreak LINeages) and pangoLEARN (version 2020-02-18) [12].

To construct a phylogenetic tree, 164 Pakistan SARS-CoV-2 sequences and their closely-related publicly-released sequences, as well as lineage representative sequences, were used. Multiple sequence alignment was performed with MUSCLE v3.8.31 [37], and the UTRs of all sequences were truncated based on nucleotide coordinates of the reference genome (GenBank: MN908947.3) [38]. A maximum likelihood phylogeny was then inferred under the best-fit model of nucleotide substitution ‘GTR+F+I+G4’ in IQ-TREE [39], [40], and tree topology was assessed with the fast bootstrapping function with 1000 replicates.

### Estimation of virus introductions

The potential introduction events were estimated as the sum of the number of virus lineages (viruses sharing the same MRCA) and the number of samples with known foreign travel histories.

## Ethical statement

The authors certify that written informed consent has been obtained from all the participating subjects.

## Data availability

The raw sequencing data and the whole genome sequences reported in this study have been deposited in the Genome Sequence Archive (GSA) [41] and Genome Warehouse (GWH) [42] in the National Genomics Data Center [43], Beijing Institute of Genomics, Chinese Academy of Sciences / China National Center for Bioinformation (GSA: CRA003122; GWH: GWHAOJE01000000–GWHAOOX01000000) that are publicly accessible at https://ngdc.cncb.ac.cn/.

## CRediT author statement

**Shuhui Song:** Conceptualization, Methodology, Writing - original draft. **Cuiping Li:** Methodology, Formal analysis. **Lu Kang:** Methodology, Formal analysis, Writing - original draft. **Dongmei Tian:** Methodology, Formal analysis. **Nazish Badar:** Resources. **Wentai Ma:** Methodology, Formal analysis. **Shilei Zhao:** Methodology, Formal analysis. **Xuan Jiang:** Methodology. **Chun Wang:** Methodology. **Yongqiao Sun:** Methodology. **Wenjie Li:** Methodology. **Meng Lei:** Methodology. **Shuangli Li:** Methodology. **Qiuhui Qi:** Methodology. **Aamer Ikram:** Resources. **Muhammad Salman:** Resources. **Massab Umair:** Investigation. **Huma Shireen:** Investigation. **Fatima Batool:** Investigation. **Bing Zhang:** Methodology. **Hua Chen:** Methodology. **Yun-Gui Yang:** Methodology. **Amir Ali Abbasi:** Investigation. **Mingkun Li:** Conceptualization, Supervision, Writing - review & editing. **Yongbiao Xue:** Conceptualization, Supervision. **Yiming Bao:** Conceptualization, Supervision, Writing - review & editing. All authors have read and approved the final manuscript.

## Competing interests

The authors declare that they have no competing interests.

## Supplementary material

Supplementary File S1 **Selective pressures during the intra-host evolution of SARS-CoV-2**

Supplementary Figure S1 **Epidemic in Pakistan** Distribution of daily confirmed cases, deaths, and detections in Pakistan as of October 9, 2020. The dates marked in red represent the date for the first reported cases, the date when Pakistan announced the upgrading of prevention and control, and announced the closure of all international flights, respectively.

Supplementary Figure S2 **Sequencing coverage and genomic variants of SARS-CoV-2 genome sequences in Pakistan** A. Distribution of sequencing depth, percentage of reference sequence covered, and number of bases covered > 30×. B. Correlations between SARS-CoV-2 read count, genome coverage, and Ct value. The number of SARS-CoV-2 reads (RPM) for all samples, proportion of the genome with sequencing depth ≥ 1 and ≥ 100, and the Ct value below the detection limit (39) was replaced by a Ct value of 42 for better visualization. Spearman’s rank correlation coefficient and p value are shown in the figure. C. Distribution of sequence count for different number of variants. Sequences from Pakistan with different MAFs and publicly-released sequences at two different dates were analyzed. RPM, reads per million.

Supplementary Figure S3 **The haplotype network and phylogenetic analysis of SARS-CoV-2 genome sequences with the collection date before June 17, 2020** Totally 164 viruses sampled from Pakistan (150 in current study and 14 publicly-released; colored in red) and 2135 randomly sampled high-quality publicly-released global sequences from other countries (from 54,204 sequences with the collection date before June 17, 2020) were included. A. Haplotype network. B. Phylogenetic tree.

Supplementary Figure S4 **Comparison of the epidemic development between Pakistan, its neighboring countries, and putative importing countries**

Supplementary Figure S5 **Evolution and transmission analysis of SARS-CoV-2 genome sequences as of February 22, 2021** Haplotype network of all SARS-CoV-2 sequences in Pakistan (Pakistan; red node), publicly-released sequences with G8371T mutation (G8371T mutated; orange node), and the closely-related sequences from other countries (Others; blue node) as of February 22, 2021.
