# Supplementary material for: Genomic Epidemiology of SARS-CoV-2 in Pakistan
Source: Genomics Proteomics Bioinformatics. 2021 Oct 23;19(5):727–40. doi: 10.1016/j.gpb.2021.08.007 (PMC8546014; doi:10.1016/j.gpb.2021.08.007)
Supplement: Supplementary File S1 — Selective pressures during the intra-host evolution of SARS-CoV-2 [file mmc1.docx]

**File S1 Selective pressures during the intra-host evolution of SARS-CoV-2**

The overall Ka/Ks ratio was 0.36, indicating a purifying selection on the viral genome (*P* < 0.001, Fisher’s exact test), which is also true for individual genes including *ORF1a*, *ORF1b*, *S*, and *M*. Notably, all these genes showed similar pattern in the population data as of September 11, 2020 (Figure S6). *ORF3a* showed a significant signature of positive selection with Ka/Ks = 6.83, which was mostly (57/76) attributed to the non-synonymous mutations occurring at two hypermutable positions in this gene, namely 25406 and 25563.


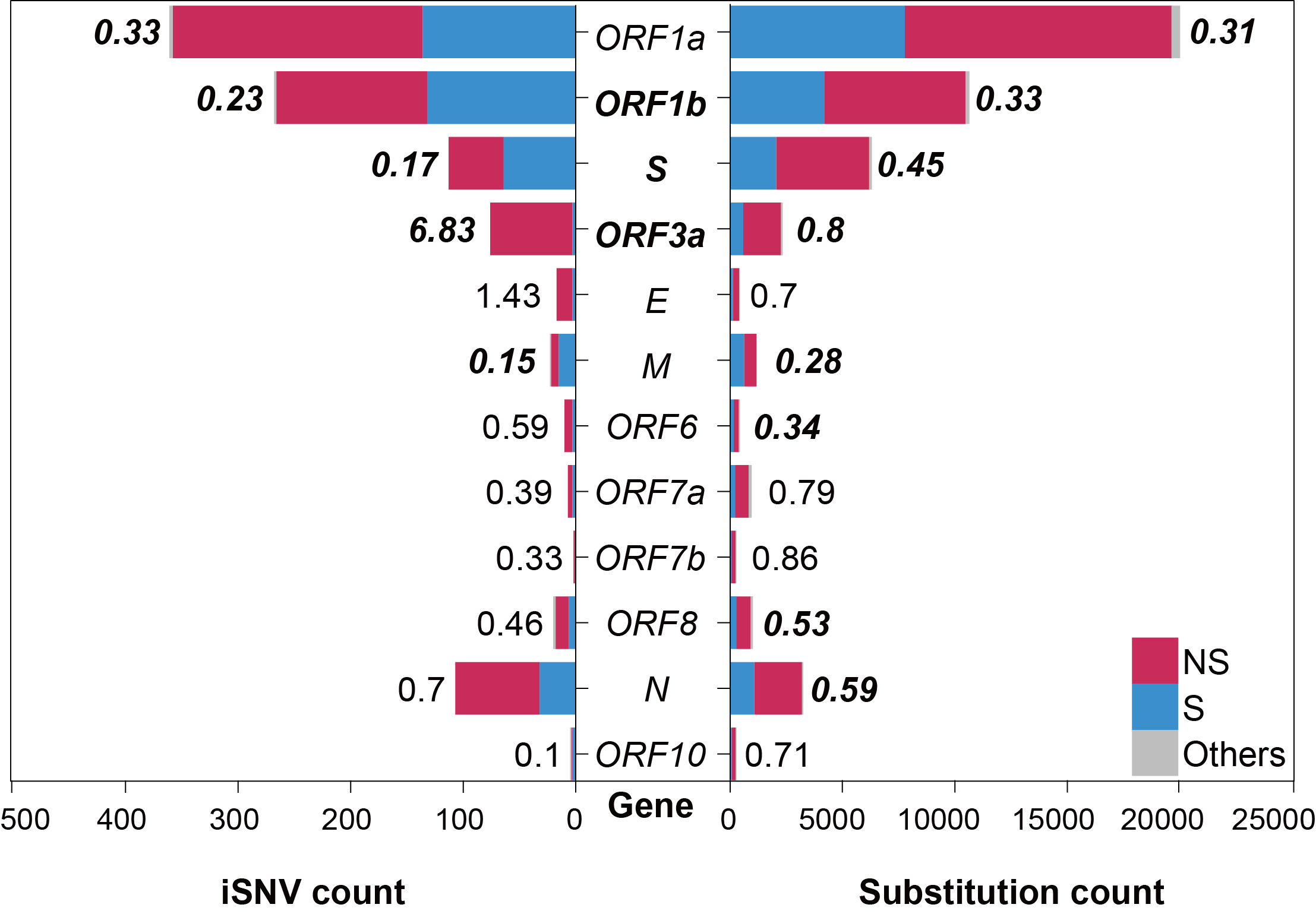


**Figure S6 Ka/Ks ratio for each gene using the iSNV data and the polymorphism data**

Ka/Ks ratios were calculated using KaKs_Calculator2.0 software (MS model) [1]. Values significantly different from 1 are indicated with italics in bold, names of genes with Ka/Ks ratios significantly different between the iSNV data and polymorphism data are presented in bold.

To further determine which sites in *ORF3a* have undergone strong selection pressure, we used the site model of M8 in EasyCodeML (version 1.4) [2] to detect positive selection sites. Our result revealed two positive selection sites at the amino acid level, *i.e.*, M5T (Ka/Ks = 9.1 ± 3.48, genomic position: 25405–25407) and Q57H in *ORF3a* (Ka/Ks = 6.9 ± 4.8, genomic position: 25561–25563), but with no significant effect. In addition, position 25406 was prone to mutation in the 150 Pakistani individuals, but no substitution was observed in the polymorphism data (Figure 3C), suggesting that the mutation was unlikely to be favored by natural selection. In contrast, G to T mutation at position 25563 (G25563T) defined a major branch (GH clade) and occurred 54 times on the phylogenetic tree of SARS-CoV-2 genomes, and its substitution rate was ranked the 8^th^ among all genomic positions. However, it was insufficient to infer a positive selection at position 25563 based on the data available, more analysis and especially experimental validation are needed.

**References**

[1] Wang D, Zhang Y, Zhang Z, Zhu J, Yu Y. KaKs_Calculator 2.0: a toolkit incorporating gamma-series methods and sliding window strategies. Genomics Proteomics Bioinformatics 2010;8:77–80.

[2] Gao F, Chen C, Arab DA, Du Z, He Y, Ho SYW. EasyCodeML: a visual tool for analysis of selection using CodeML. Ecol Evol 2019;9:3891–8.
