# Supplementary figures and images for: Genomic Epidemiology of SARS-CoV-2 in Pakistan
Source: Genomics Proteomics Bioinformatics. 2021 Oct 23;19(5):727–40. doi: 10.1016/j.gpb.2021.08.007 (PMC8546014; doi:10.1016/j.gpb.2021.08.007)

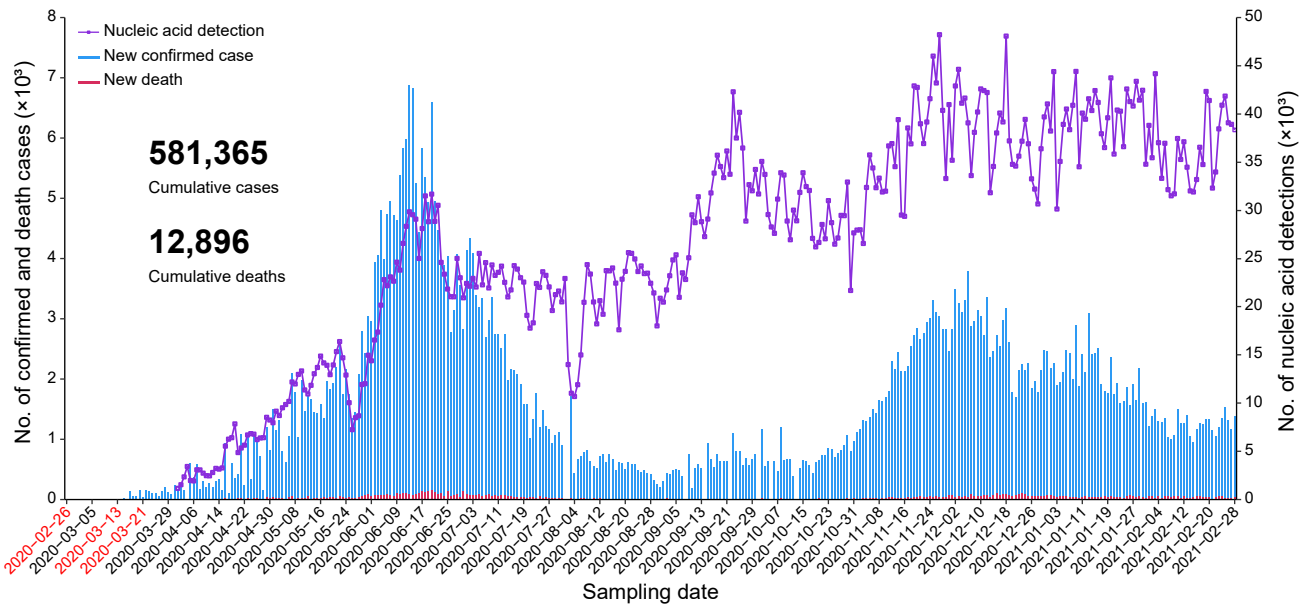

Supplement: Supplementary Figure S1 — Epidemic in Pakistan Distribution of daily confirmed cases, deaths, and detections in Pakistan as of October 9, 2020. The dates marked in red represent the date for the first reported cases, the date when Pakistan announced the upgrading of prevention and control, and announced the closure of all international flights, respectively. [file mmc2.pdf]

**A**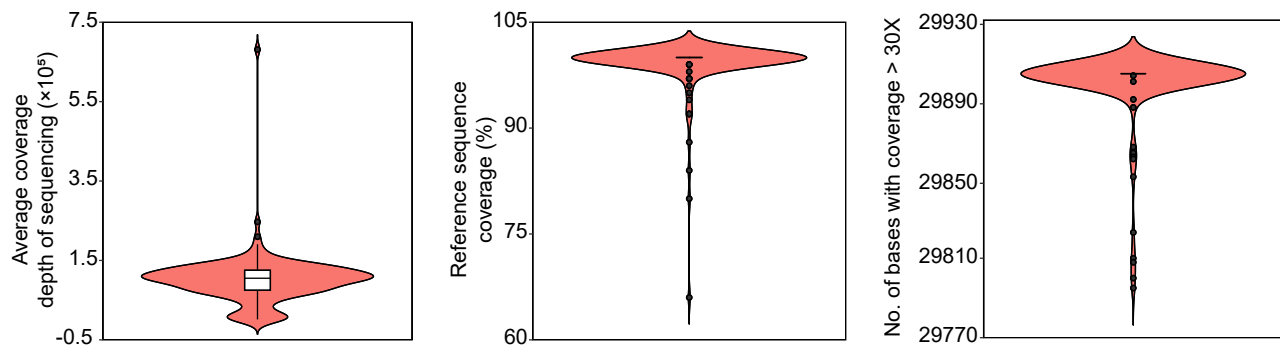**B**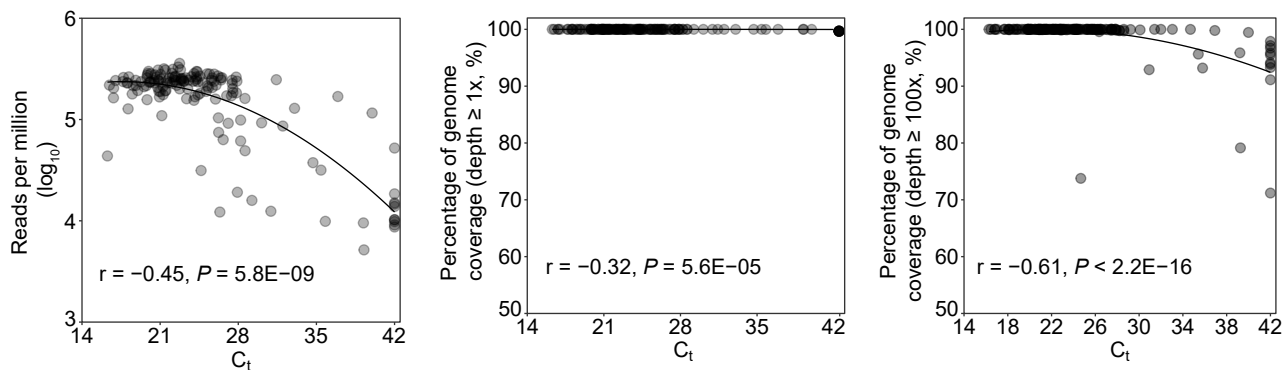**C**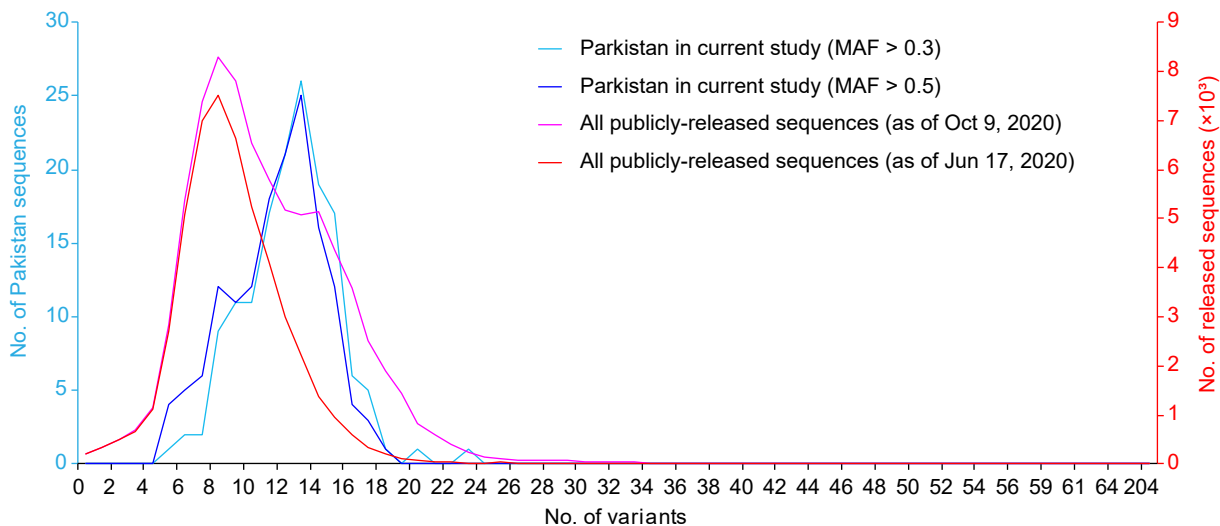

Supplement: Supplementary Figure S2 — Sequencing coverage and genomic variants of SARS-CoV-2 genome sequences in Pakistan A. Distribution of sequencing depth, percentage of reference sequence covered, and number of bases covered > 30×. B. Correlations between SARS-CoV-2 read count, genome coverage, and Ct value. The number of SARS-CoV-2 reads (RPM) for all samples, proportion of the genome with sequencing depth ≥ 1 and ≥ 100, and the Ct value below the detection limit (39) was replaced by a Ct value of 42 for better visualization. Spearman’s rank correlation coefficient and p value are shown in the figure. C. Distribution of sequence count for different number of variants. Sequences from Pakistan with different MAFs and publicly-released sequences at two different dates were analyzed. RPM, reads per million. [file mmc3.pdf]

**A**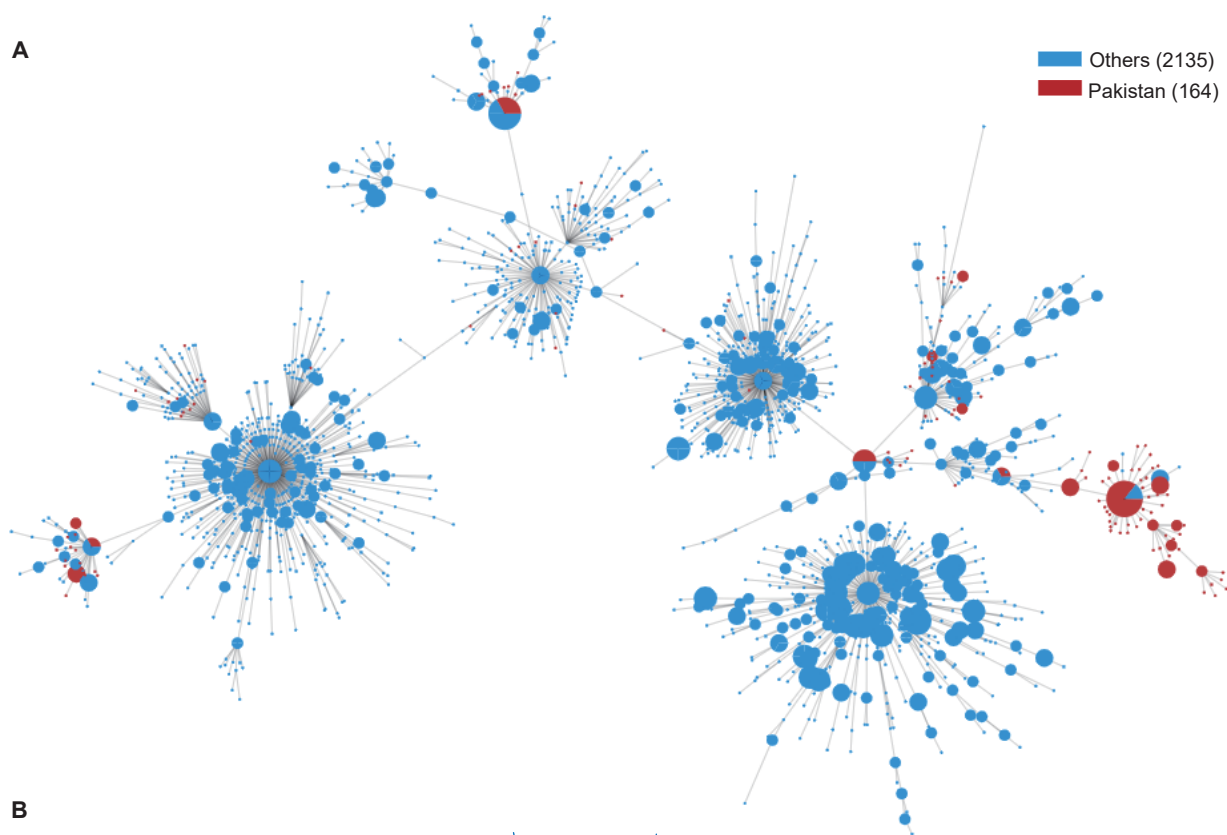**B**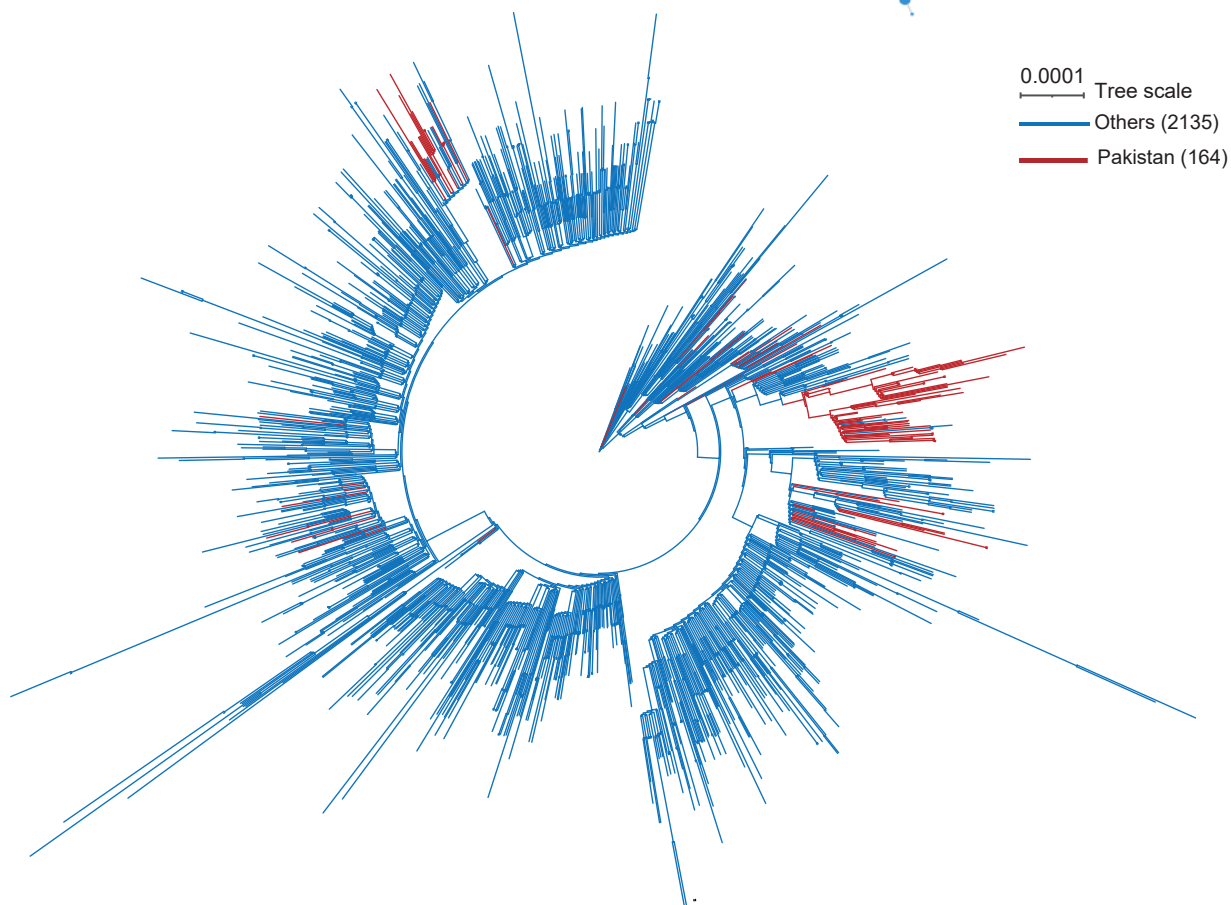

Supplement: Supplementary Figure S3 — The haplotype network and phylogenetic analysis of SARS-CoV-2 genome sequences with the collection date before June 17, 2020 Totally 164 viruses sampled from Pakistan (150 in current study and 14 publicly-released; colored in red) and 2135 randomly sampled high-quality publicly-released global sequences from other countries (from 54,204 sequences with the collection date before June 17, 2020) were included. A. Haplotype network. B. Phylogenetic tree. [file mmc4.pdf]

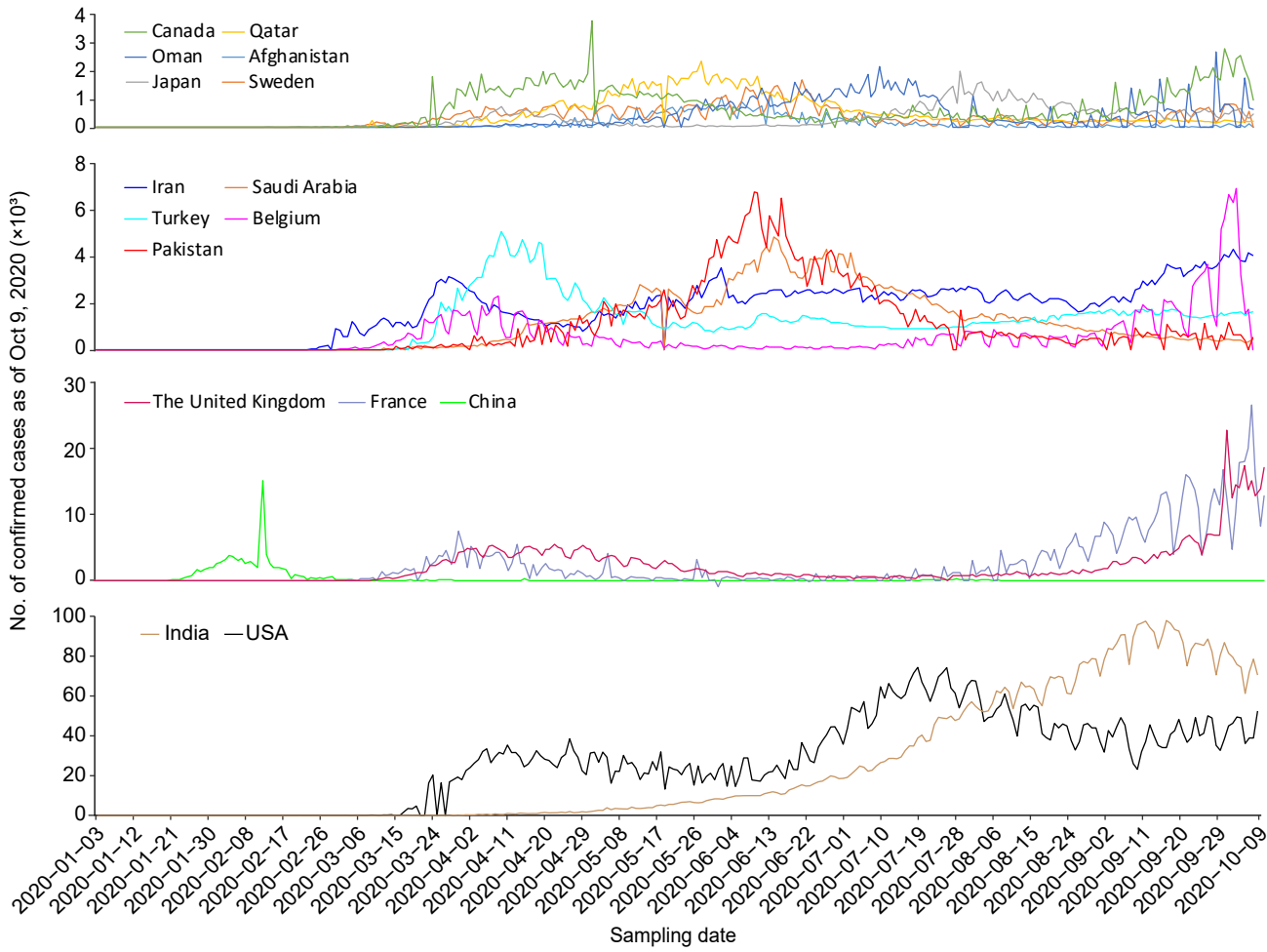

Supplement: Supplementary Figure S4 — Comparison of the epidemic development between Pakistan, its neighboring countries, and putative importing countries [file mmc5.pdf]

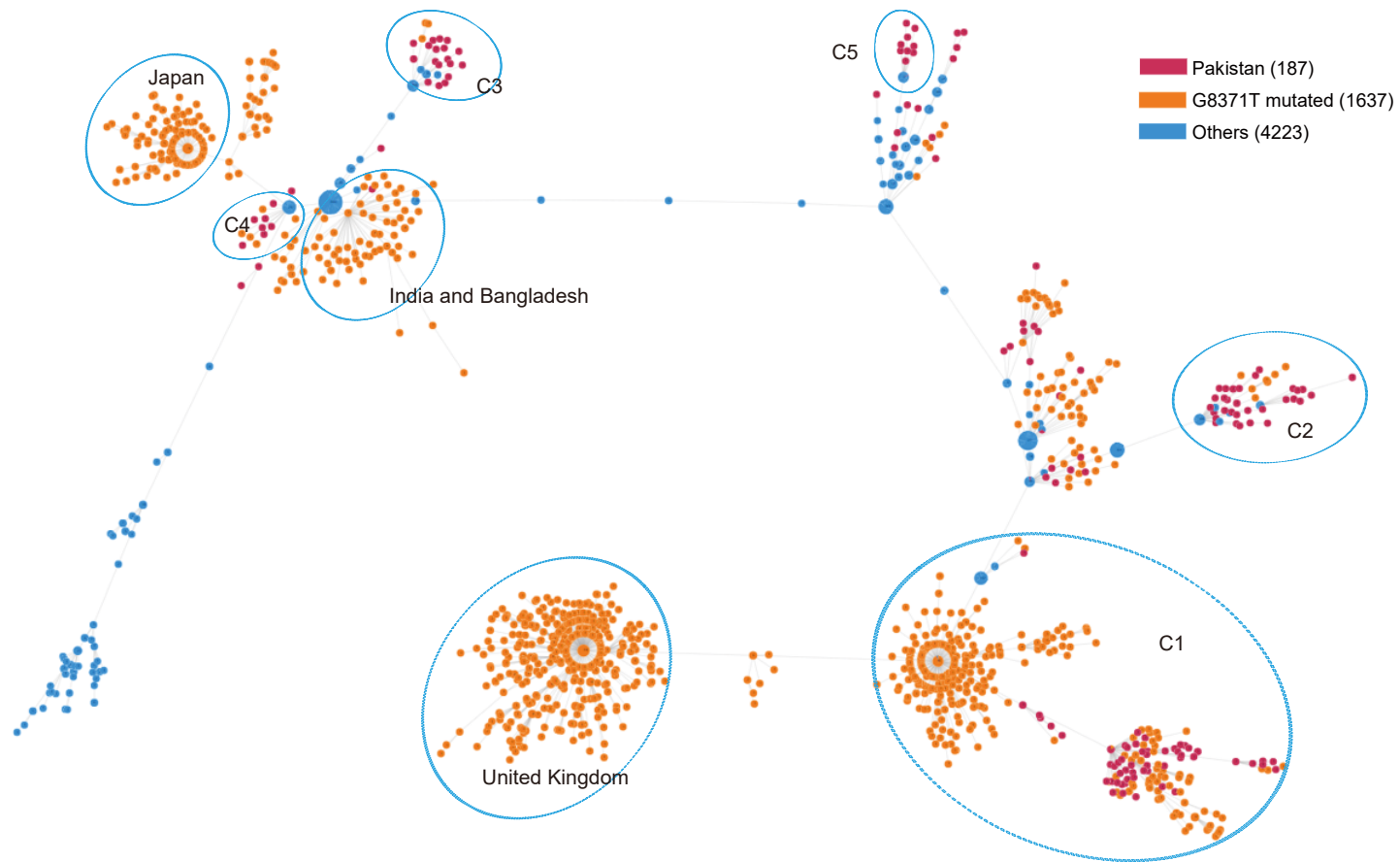

Supplement: Supplementary Figure S5 — Evolution and transmission analysis of SARS-CoV-2 genome sequences as of February 22, 2021 Haplotype network of all SARS-CoV-2 sequences in Pakistan (Pakistan; red node), publicly-released sequences with G8371T mutation (G8371T mutated; orange node), and the closely-related sequences from other countries (Others; blue node) as of February 22, 2021. [file mmc6.pdf]
